# The Fluid Mechanics of Ureteroscope Irrigation

**DOI:** 10.1089/end.2018.0707

**Published:** 2019-01-18

**Authors:** Jessica G. Williams, Benjamin W. Turney, Niraj P. Rauniyar, Timothy P. Harrah, Sarah L. Waters, Derek E. Moulton

**Affiliations:** ^1^ Mathematical Institute, University of Oxford, Oxford, United Kingdom.; ^2^ Nuffield Department of Surgical Sciences, University of Oxford, John Radcliffe Hospital, Oxford, United Kingdom.; ^3^ Department of Urology and Pelvic Health, Boston Scientific Corporation, Marlborough, Massachusetts.

**Keywords:** benchtop experiments, mathematical modeling, optimization, pipe flow

## Abstract

***Purpose:*** To develop a physical understanding of ureterorenoscopy irrigation, we derive mathematical models from basic physical principles and compare these predictions with the results of benchtop experiments. Mathematical modeling can be used to understand the role of inlet pressure, tip deflection, the presence of working tools, geometric properties of the instruments used, and material properties of the irrigation fluid on resulting flow rate.

***Materials and Methods:*** We develop theoretical models to describe irrigation flow in an idealized setup and compare with benchtop experiments for flow through a straight scope, a scope with a deflected tip, and a scope with a working tool inserted. The benchtop experiments were performed using Boston Scientific LithoVue ureteroscope and a variety of Boston Scientific working tools. Standard ureteroscope working channels have circular cross sections, but using theoretical models we investigate whether modifications to the cross-sectional geometry can enhance flow rates.

***Results:*** The theoretical flow predictions are confirmed by experimental results. Tip deflection is shown to have a negligible effect on flow rate, but the presence of working tools decreases flow significantly (for a fixed driving pressure). Flow rate is predicted to improve when tools are placed at the edge of the channel, rather than the center, and modifying the cross-sectional shape from a circle to an ellipse can further increase flow rate.

***Conclusions:*** A mathematical framework is formulated and shown to accurately predict the properties of ureteroscope irrigation flow. The theoretical approach has significant potential in quantifying irrigation flow and improving ureteroscope design.

## Introduction

Flexible ureterorenoscopy provides a minimally invasive treatment for the destruction and removal of kidney stones. The procedure is performed by passing auxiliary working tools (e.g., laser fibers and baskets) through the working channel of a ureteroscope, and utilizing the actively deflectable tip. An ongoing challenge is to optimize visualization within the renal collecting system during treatment. Good intrarenal views are obtained by flowing irrigation fluid (saline) through the scope to create a working space within the kidney and to clear this region of debris from stone fragmentation or blood. There are several different ways that urologists deliver and drain the irrigation fluid, but a typical approach is to connect a bag of saline to the scope inlet via irrigation tubing. The height at which the bag is hung affects the driving pressure at the scope inlet and subsequent inflow and outflow rates. The flow is complicated by working tools of varying sizes inserted into the channel of the ureteroscope, and there is no systematic procedure for varying the flow or quantifying the relationship between flow and intrarenal pressure.

To understand the impact of procedural modifications in a clinical setting, various experimental studies have previously been conducted exploring the relationship between saline bag height, instrument size, tip deflection, and flow rate, and their potential impact on visualization. Flow rate was found to increase, although nonlinearly, with height in cystoscopy irrigation.^2^ Working tool size has been shown to have a considerable effect on the flow of irrigation fluid and subsequent visibility, while deflection of the scope tip did not seem to impact these metrics significantly.^3–8^ The notable decrease in irrigation flow caused by working tools in particular has led to experimental exploration of ureteroscope design modifications, such as the addition of a second working channel^9^ or procedural adjustments such as removing the coating on nitinol stone baskets.^10^

While such experimental studies provide useful empirical evidence, they are limited both by a sparsity of data and a lack of theoretical underpinning. Thus, the potential for mathematical modeling approaches to advance the field is significant. A mechanistic mathematical model can provide fundamental insights into system behavior. Simulations can be performed across multidimensional parameter spaces that are too time-consuming, costly, and/or impractical to access via experiment. The resulting quantitative predictions can be exploited to assess the implications of ureteroscope geometry (e.g., length, diameter, cross-sectional shape) and operating conditions (e.g., saline bag height, irrigation fluid properties) on the resulting fluid flows, and facilitate the optimization of ureteroscope design and clinical protocols.

Our objective in this article is to develop and validate a mathematical model for fluid flow through ureteroscopes to investigate the impact of scope and working tool geometries as well as operating conditions on the resulting irrigation flows. We consider an idealized model for flow through the working channel, with the governing equations derived via systematic reductions of the continuity and Navier-Stokes equations of fluid mechanics. We also perform benchtop experiments to complement the mathematical analysis. Model predictions for the fluid flow rate are validated by comparison with our experimental data in three distinct settings: (I) a straight scope, (II) a scope with deflected tip, and (III) a scope with working tool inserted. We demonstrate a strong dependence of flow rate on the size of the working tool as well as its location within the working channel, relationships that are made mathematically explicit. We then use the model to investigate the effect of a noncircular cross-sectional geometry; in particular, we identify an elliptical channel shape that optimizes flow rate for a given channel cross-sectional area and inlet pressure.

## Materials and Methods

### Experiments

**FIG. 1.**
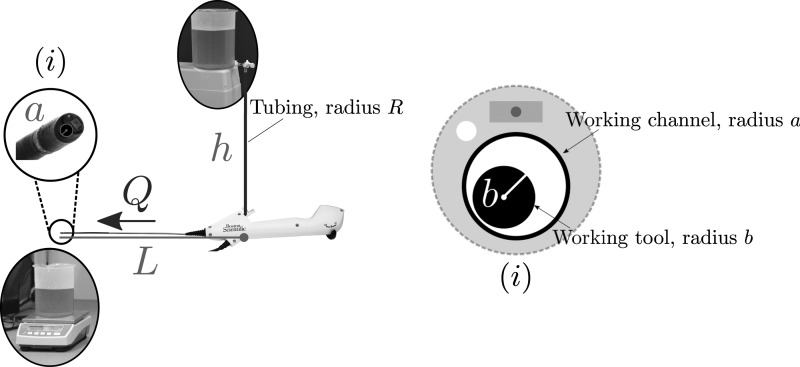
A schematic of the experimental setup labeled with variables and parameters used in the theoretical model. The top of the fluid is at height *h* above the scope and flows through tubing of radius *R*. The ureteroscope working channel is of radius *a* and length *L*. The flow rate, *Q*, is measured using a mass balance. *Inset* figure **(i)** demonstrates a cross-sectional view of the scope, with the working channel containing a working tool of radius *b*.

A schematic of the experimental setup used to measure flow through a Boston Scientific LithoVue ureteroscope is pictured in Figure 1. Details on the experimental setup and data analysis are provided in Supplementary Data (Supplementary Data are available online at www.liebertpub.com/end). Three forms of experiment were performed:

I. A *Straight Scope* with no working tool but varying inlet pressure.
II. A scope with *Deflected Tip* of varying degree.
III. A straight scope with *Working Tools* of varying size inserted.

### Theoretical modeling

We consider irrigation flow in an idealized setting with fluid at fixed inlet pressure entering a horizontal scope, whose distal end is taken to be at ambient pressure. Mathematical details are given in the Mathematical Modeling section of Supplementary Data.

## Results

### I. Straight scope

For a straight scope with no tools, the volumetric flow rate, *Q*, of fluid through the ureteroscope is described by Poiseuille flow.^11^ For a circular cross section of radius *a* and length *L*, this reads

\begin{align*}
Q = { \frac { { a^4 } \pi \Delta P }  { 8 \mu L } }   \tag { 1 }
\end{align*}

where $\Delta P$ is the difference between the inlet and outlet pressures and $\mu$ is the dynamic viscosity of the irrigation fluid.

**FIG. 2.**
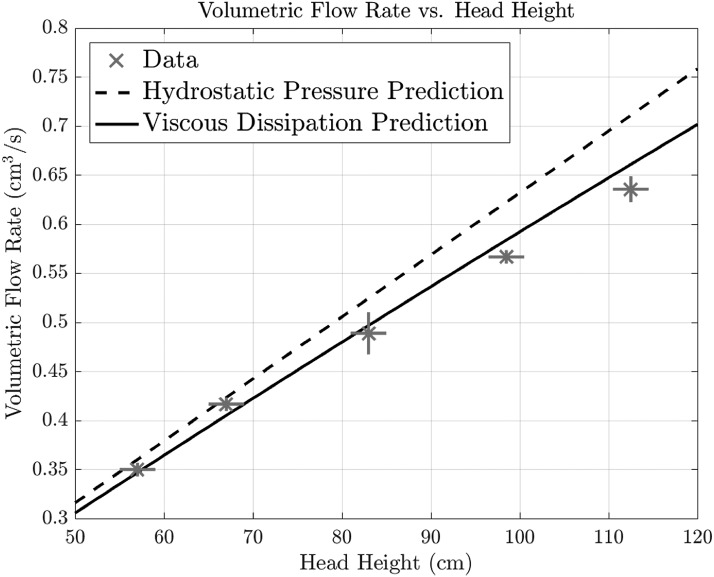
Experimental and theoretical results for the flow of water through an open ureteroscope working channel of internal radius 0.06 cm. The *dashed line* [Equation (1)] assumes a hydrostatic inlet pressure. The *solid line* [Equation (2)] accounts for viscous dissipation. *Horizontal lines* on the data points depict error in the height measurements, and *vertical lines* depict error in measured flow values.

In a given surgical setting, *a*, *L*, and $\mu$ are fixed and hence Equation (1) is best viewed as a relationship between flow rate and pressure drop. For a hydrostatic pressure head, $\Delta P = \rho gh$, where $\rho$ is fluid density, *g* is gravitational acceleration, and *h* is the height of the hydrostatic column of fluid, measured from the point where the irrigation tubing enters the scope (Fig. 1). This theory predicts a linear relationship between flow rate and head height, which is plotted as the dashed line in Figure 2. Compared with the experimental values for a straight scope (the data points in Fig. 2), we see a systematic deviation between this linear prediction and experiment, which becomes more pronounced at higher head heights. This deviation from the linearity predicted by the classic Poiseuille result was also observed in a previous study on irrigation in cystoscopy.^2^

We propose that the discrepancy arises from neglecting the flow that occurs from the suspended reservoir into the scope. Including the vertical flow through the irrigation tubing of radius *R* in our model incorporates the effect of viscous dissipation, and results in an inlet pressure that is lower than the hydrostatic approximation. We thus obtain the modified pressure relationship

\begin{align*}
\Delta P = \rho gh \left( { 1 - \frac { \sigma }  { { 1 + \sigma } } } \right) ,  \tag { 2 }
\end{align*}

where $\sigma = \left( { \frac { h }  { L } } \right) { \left( { \frac { a }  { R } } \right) ^4 }$ characterizes the relative importance of viscous dissipative effects. As *h* increases (or *R* decreases), $\sigma$ will increase and the imposed pressure difference is increasingly smaller than $\rho gh$. In Figure 2, the model prediction with $\Delta P$ specified by Equation (2) is shown as the solid line, which indeed shows superior agreement with the experiment.

For the parameters explored here, viscous dissipation becomes increasingly significant as the height of the irrigation fluid increases (Fig. 2). We thus conclude that even in this simple setting, classic results may yield inaccurate predictions. In a clinical setting, the dependence of the volumetric flow rate on both height and tube radius should be considered when determining how high to hang the irrigation bag.

### II. Deflected tip

**FIG. 3.**
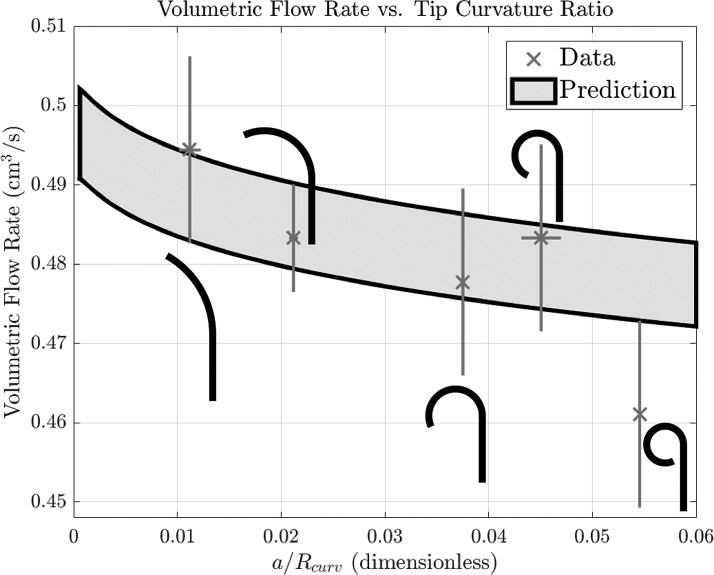
Experimental and theoretical results for flow through an open channel with a deflected tip. The *horizontal axis* shows the ratio of the radius of the working channel (0.06 cm) to the radius of curvature of the curved tip. *Inset diagrams* demonstrate the extent of tip deflection for each data point. Experiments performed at $h = 83$ cm (uncertainty in this measurement applied to theory provides *gray* region). *Vertical* error bars on experiments depict error in flow measurements, and *horizontal* error bars provide uncertainty in radius of curvature measurements. (When the tip is maximally curved, the channel outlet is partially blocked by the scope shaft, which may explain the flow rate falling below predictions in the final data point.)

We next consider the effect of ureteroscope tip deflection on flow rate. The ureteroscope shaft is comprised of a straight channel (73.3 cm long) connected to a shorter deflectable tip (5.7 cm). By translating classic results on Dean flow^12^ into our setting, and connecting flow through the curved tip with a Poiseuille description through the straight portion, we obtain flow rate as a function of tip curvature and previous parameters. Figure 3 plots flow rate against tip curvature. Experimental measurements are plotted against the theoretical prediction, appearing as the gray region and incorporating measurement uncertainty in the height of the fluid above the scope (and hence the inlet pressure). The horizontal axis is the dimensionless curvature, with a larger value corresponding to greater deflection. We find a relatively minimal decrease in flow rate with tip deflection. For example, the highest possible LithoVue curvature corresponds to only a 5% decrease in flow rate compared with that of a straight scope. This confirms observations from previous studies,^3,4,8^ while providing a theoretical underpinning.

### III. (a) Working tool results (circular channel)

We next consider the effect of working tools. If a tool of circular cross section with radius *b* lies concentrically within the channel (of radius *a*), the flow rate can be computed analytically, leading to the following adapted form of Equation (1):

\begin{align*}
Q = { \frac { \pi \left( { { a^2 } - { b^2 } } \right) \Delta P }  { 8 \mu L } } \left[ { { a^2 } + { b^2 } - { \frac { { a^2 } - { b^2 } }  { \log \frac { a }  { b } } } } \right]. \tag { 3 }
\end{align*}

**FIG. 4.**
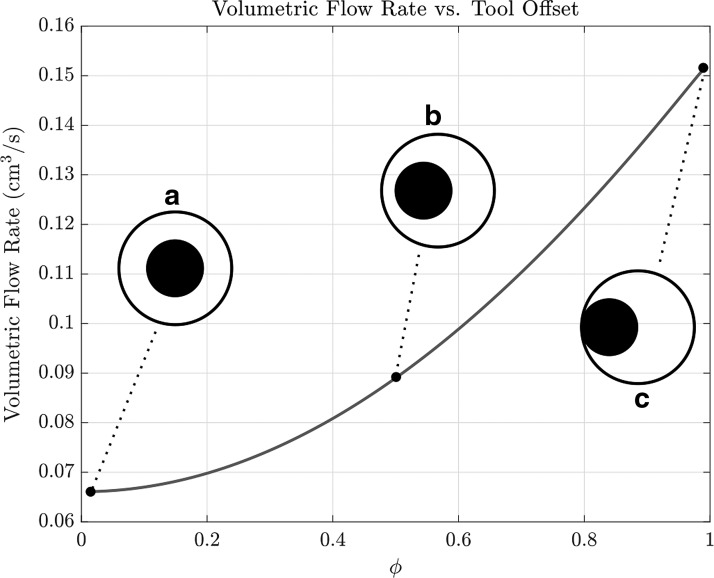
The theoretical flow rate with hydrostatic driving pressure $\Delta P = \rho gh$, for $h = 83$ cm, is plotted against $\phi$, which gives a measure of how offset the tool is within the channel. These predictions are for a channel of radius $a = 0.06$ cm and working tool of radius $b = 0.03$ cm. The *inset* schematics correspond to (a) $\phi = 0.01$, (b) $\phi = 0.5$, and (c) $\phi = 0.99$.

The flow rate depends nonlinearly on the size of the tool, so small increases in tool radii lead to large decreases in flow. In Poiseuille flow (no tool), the axial fluid velocity is greatest at the center of a circular cross section. Hence, Equation (3) provides a lower bound on the flow rate, as any deviation to the position of the working tool from the center should create less resistance. To explore the effect of tool position, we define a parameter $\phi$ that denotes the degree of offset; $\phi = 0$ (center) and $\phi = 1$ (edge). Figure 4 plots flow rate as a function of $\phi$, showing a significant increase in flow rate for a working tool at the edge of the channel, compared with the center.

**FIG. 5.**
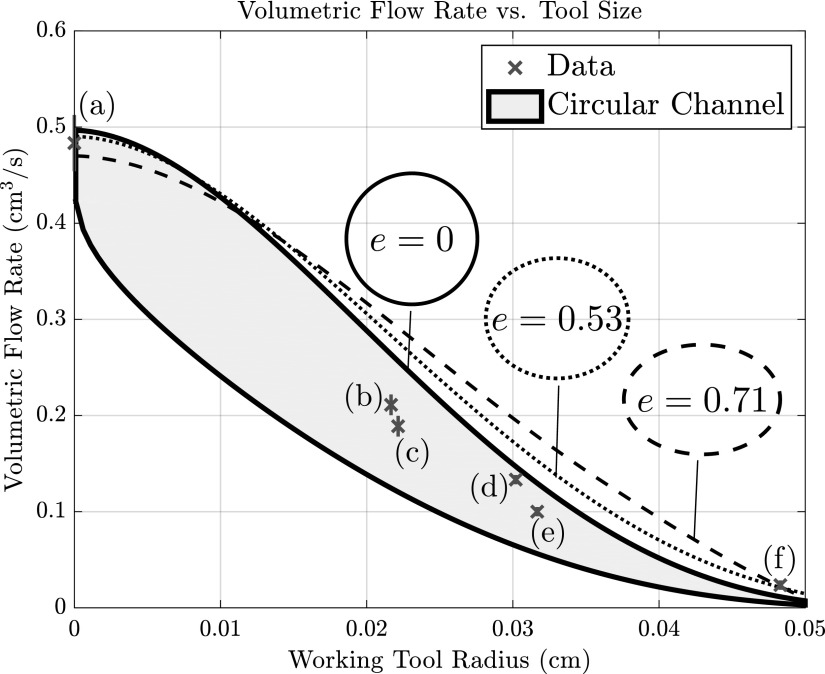
Experimental and theoretical results for flow through a working channel containing a working tool with a column of irrigation fluid at height $h = 83$ cm. The *gray* region indicates flow predictions for a circular working channel; the *lower line* for a tool concentric within the working channel [Equation (3)], and the *upper line* for a tool at the edge. Each data point corresponds to the experimentally measured flow rate when a different Boston Scientific working tool is within the working channel. *Vertical* error bars on the experimental data give the error in measured flow rates. The tools used were (a) No tool, (b) OptiFlex, (c) Flexiva 200, (d) Flexiva 365, (e) Zero Tip, (f) ZIPwire. The *dashed* and *dotted lines* show the maximum (over all possible tool positions) theoretical predictions for elliptical working channels of the shapes specified by the corresponding *inset* schematics. The labeled *e* refers to the eccentricity of the theoretical elliptical working channel shapes. Note that tool (f) created an extremely low flow, exiting the scope in a series of drips, rather than the steady stream assumed by the mathematical model; this may account for the discrepancy in the predicted flow.

In practice, the position of the tool within the working channel is unknown, so we expect flow measurements to lie within these minimal and maximal values. Figure 5 plots flow rate as a function of working tool radius; the gray region indicates the theoretical range of flow for a circular working channel. The data points correspond to experimentally measured flow rates for different working tools.

### III. (b) Noncircular working channel

We have demonstrated the basic physical principle that the flow resistance created by the presence of a working tool is strongly tied to the geometry of the space available for fluid flow. This motivates the question of whether a more optimal channel configuration could be realized with a noncircular working channel cross section. To pose a well-defined mathematical problem, we fix the cross-sectional area of the channel and seek a geometry—both channel shape and tool position—that optimizes the flow, in the sense of maximizing the flow rate for given inlet pressure. Furthermore, for both mathematical and potential manufacturing simplicity, we restrict our analysis to elliptical shapes for the working channel cross section.

Subject to these constraints, we varied the elliptical shape, characterized by its eccentricity (a parameter 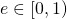), which measures how elongated the ellipse is, where $e = 0$ describes a circle). We also varied the position of the tool within the elliptical channel and simulated the resulting flow.^13^ For an unobstructed channel, a circular cross section provides higher flow than an elliptical one. With a tool inserted, however, and considering the tool position corresponding to maximal flow rate, an elliptical channel can lead to significantly higher flow rates. We refer back to Figure 5, which also includes the predicted flow rates through elliptical channels for two eccentricities (dashed line, $e \approx 0.71$; dotted line $e \approx 0.53 )$. The position of the tool in the elliptical channel is taken to be the one that produces the maximum flow; this is the position as close to the edge of the channel as possible for a given tool. We find that for working tools with a radius smaller than ∼0.01 cm, the circular channel provides greater flow than the two ellipse geometries. As the working tool size is increased, however, the maximum flow occurs with an elliptical geometry.

**FIG. 6.**
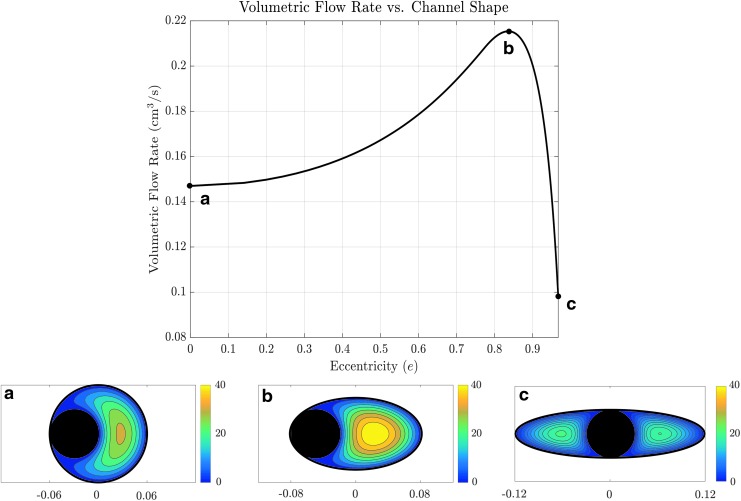
Theoretical results for flow as a function of ellipse eccentricity for a driving pressure calculated assuming an 83 cm head height, accounting for the effects of viscous dissipation. The working tool has a 0.604 mm diameter, and working channel cross-sectional area constrained to be that of a circular channel with a 1.20 mm diameter. *Inset* figures **(a–c)** demonstrate pointwise velocity color maps for the configurations that correspond to the labeled points on the graph. The color maps give velocity in units of cm/s, and units on the *x*-axis of the *inset* plots are in cm.

This result suggests the possibility of design optimization via noncircular geometries. As a further proof of concept, we computed the irrigation flux and pointwise velocity of the fluid across the cross section of a circular working channel with diameter 1.20 mm, with a Flexiva 365 laser fiber (diameter 0.604 mm) positioned at the (optimal) edge of the channel. The corresponding flow rate is given by data point (a) in Figure 6. Changing to an elliptical cross section and varying the eccentricity while maintaining cross-sectional area, we obtain the graph in Figure 6, which plots the predicted flow rate as a function of working channel eccentricity. This shows a clear optimal channel shape and tool position, Figure 6b, enabling a flux nearly 50% higher than the flux through the circular channel. If the eccentricity is increased past this optimal value, the tool can no longer fit at the edge of the channel, and the flow is predicted to decrease [data point (c)]. The velocity color maps in Figure 6 demonstrate that the maximum velocity within the cross section is also the largest in the optimal configuration.

## Discussion

We have developed a mathematical model of flow through a ureteroscope from fundamental physical principles. The results in Part I demonstrated the need to account for viscous dissipation in correctly quantifying the driving pressure, highlighting the need for caution when applying classic theoretical results. In Parts II and III (a), we quantified the effects of scope tip deflection and the presence of working tools on irrigation. Scope deflection alone was found to only cause a small decrease in irrigation flow, but the presence of working tools resulted in a significant hindrance. Moreover, the location of the tool in the working channel has a strong effect on flow, with maximal flow occurring when the tool is at the edge of the channel. Each of these theoretical results was validated through comparison with benchtop experiments.

Having seen that cross-sectional geometry plays a critical role in flow characteristics, in Part III (b) we explored design optimality. By considering elliptical cross sections with a fixed area, we demonstrated the presence of an eccentricity value that maximizes flow rate.

## Conclusions

The potential value of using mathematical modeling to understand and aid in design of biomedical devices should not be understated. Within endourology, similar physics-based models have been used, for instance, to guide the design of ureteral stents to minimize urine reflux.^14^ The details of irrigation flow during ureteroscopy, specifically its impact on intrarenal pressure and stone visualization and movement, are hard to determine in a clinical setting. Without a direct measure of flow rate or explicit relationships between system variables, maneuvers to modify the flow rate must typically be formed in an *ad hoc* manner as deemed necessary by the operating surgeon. Mathematical modeling can improve the design of endourologic tools by providing quantitative predictions of irrigation flow and intrarenal pressure.

In this study, we examined the impact of various geometrical factors in irrigation flow with a key result: the identification of an optimal cross-sectional geometry for irrigation flow. However, it should be noted that this requires a clear and consistent definition of “optimal,” which may not be so straightforward in ureterorenoscopy. Our approach has been to view optimality as obtaining a maximal irrigation flow within the class of elliptical cross sections of fixed area, and for a given pressure head; this follows from the basic premise that increased flow will create enhanced visibility. Nevertheless, other considerations will come into play, including the effect of increased flow on kidney deformation, intrarenal pressure, and retropulsion of the kidney stone due to imposed flow. For instance, it is important not to elevate the intrarenal pressure, as most urologists believe that operating at lower renal pressures results in lower risks of renal damage, absorption of fluid and bacteria, and resulting sepsis.^1^ Such factors are the subject of ongoing work in our group. In any case, the benefit of a theoretical and computational framework is evident, as it provides an immediate estimate of flow characteristics under given assumptions.

## Supplementary Material

Supplemental data
