# Supplementary material for: The Fluid Mechanics of Ureteroscope Irrigation
Source: J Endourol. 2019 Jan 18;33(1):28–34. doi: 10.1089/end.2018.0707 (PMC6352503; doi:10.1089/end.2018.0707)
Supplement: Supplemental data [file Supp_Data.pdf]

Supplementary Data

Experiment Details

Basic setup

The basic experimental setup is depicted in Supplementary Figure S1a. A Boston Scientific LithoVue is clamped in a horizontal position, and tubing, connected to the scope, is fed from a water-filled bucket, within which the fluid is maintained at constant height by manual correction. The tubing is connected to the bucket via a valve to switch on and off the flow of water (Supplementary Fig. S1b). The tip of the scope is situated directly above a container (to collect the water flowing from the scope) on a mass balance (Kern PCB 6000-0 balance), which is connected to a laptop computer to record the mass of fluid in the container at specified intervals.

General experimental procedure

At the start of each experiment, the valve was opened to allow water to flow through the system. The water level in the bucket was maintained by continuously refilling to a marked height. Each experiment was performed for 60 seconds and mass measurements were recorded at 1-second intervals. The volumetric flow rate (in g/s) was approximated from the data by taking the final mass measurement divided by the total time.* Each experiment was performed at least three times. The experimental parameters are given in Supplementary Table S1.

Details for specific experiments

We consider three distinct experiments:

I. A straight scope.
II. A scope with a deflected tip.
III. A straight scope with a working tool inserted.

Straight scope experiments. The experiments on the straight scope were performed at five different head heights (57, 67, 83, 98.5, and 112.5 cm). To adjust the head height, the bucket was raised and longer tubing attached, so that the tubing remained vertical without slack for each experiment. Head heights were estimated using both a standard measuring tape and by analyzing photos of the setup, with a measurement error of ±1 cm.

Deflected tip experiments. For the deflected scope experiments, the head height was set at 83 ± 1 cm, and experiments were performed for five different deflections. Images of the scope at various degrees of deflection are shown in Supplementary Figure S2. The radius of curvature for each deflection was estimated by fitting a circle to a photo image of the deflected scope tip (Supplementary Fig. S2), taken next to a standard ruler for scale. The circle fitting was performed three times, and the standard deviation of the three radius values was taken to be the error on this measurement.

Working tool experiments. For the working tool experiments, the head height was set at 83 ± 1 cm, and different Boston Scientific working tools (Supplementary Table S2) were inserted into the working channel before starting the experimental procedure.

Compared with the case of no tool, the largest tool used, the ZIPwire, caused a 93% reduction in flow; the Flexiva 200 and 365 laser fibers led to 60% and 72% decreases in flow, respectively; and the OptiFlex and Zero Tip baskets caused 56% and 79% reductions, respectively (Fig. 5).

Data Analysis

The raw data from the experiments, obtained as mass measurements at 1-second intervals, are displayed in Supplementary Figure S3.

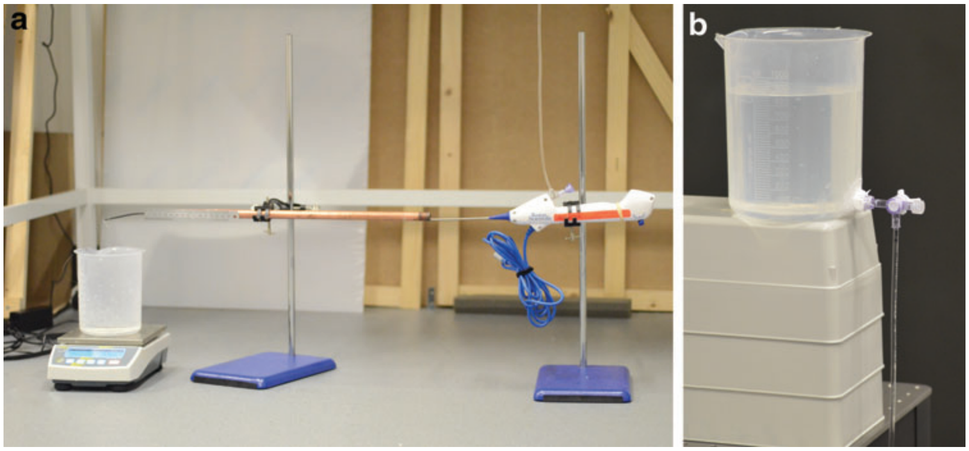

SUPPLEMENTARY FIG. S1. **(a)** A photo of the experimental setup. The LithoVue is held in position by passing it through a horizontally clamped metal tube. The inlet to the scope is attached via vertical tubing to a bucket, the height of which can be adjusted **(b)**. The distal end of the scope is open to the air, and the fluid flows from this into a second bucket on a mass balance.

*The flux through the working channel with a ZIPwire inserted (data point (*f*) in Fig. 5) was calculated differently (Data Point (*f*) in Figure 5 section).

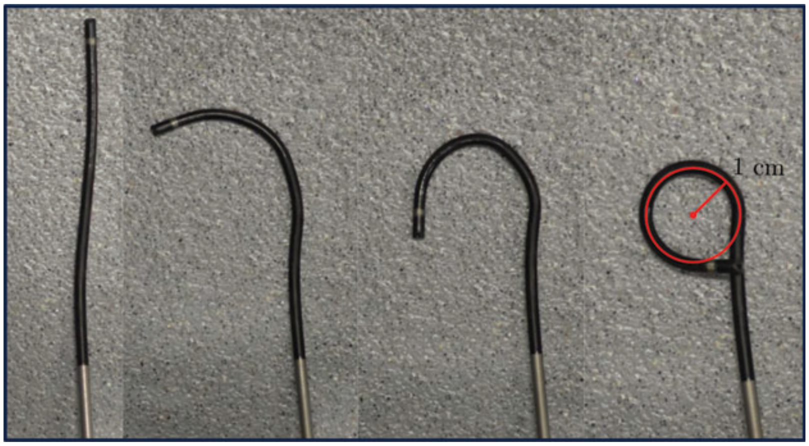

SUPPLEMENTARY FIG. S2. Boston Scientific's LithoVue at various stages of deflection. The image on the far right shows the LithoVue at its maximum deflection, corresponding to a radius of curvature of 1 cm.

Estimating flow rate

The flow rate was approximated from the data by taking the final mass measurement for each experimental run and dividing it by the total time that the run was performed for. For the working tool and deflected scope experiments, three 60-second runs were performed for each working tool or radius of curvature, respectively. For the straight scope experiments, a minimum of three 60-second runs were performed at each height. The mean flow rates, Q, and standard deviations, σ, in the estimated flow rates were recorded for every experimental setup.

Data point (*f*) in Figure 5. The raw data for the mass of fluid exiting the scope with a ZIPwire inserted is shown as light-blue lines in Supplementary Figure S3c. As the flow is so low, the increase in mass is small. The mass balance has a sensitivity of 1 g, and the mass only increased by 2 g over the 60-second interval. In this slow-flow region, we approximate flow rate by taking $Q = 1/T_{1,2}$ g/s, where $T_{1,2}$ is the time in seconds for the mass to increase from 1 to 2 g (demonstrated for one particular run by the dotted black lines in Supplementary Figure S3c.

Systematic (measurement) error

We used a mass balance Kern PCB 6000-0, which has a readability of 1 g. Therefore, the error in the final mass measurement was taken to be $\Delta M = \pm 0.5$ g, and the error in the estimated flow rate for each run to be $\sqrt{2}\Delta M/T$, where T is the total time of the experimental run. Therefore, the systematic error in the mean flow rate is $\Delta Q = \sqrt{2\sum_{i=1}^{n}(\Delta M/T)^2}$, where n is the number of runs for each configuration (ranging from 3 to 14).

Supplementary Table S1. Table of Experimental Parameters

Working channel radius (a)	0.06 cm
Working channel length (L)	79 cm
Density of water (ρ)	1 g/cm^3
Dynamic viscosity of water (μ)	0.01 g/(cm·s)
Gravitational acceleration (g)	981 cm/s^2
Length of flexible tip (L_c)	5.7 cm

Total error

We assume that the standard deviation in flow rates and the measurement error due to readability of the mass balance are independent, and thus, our total error estimation is as follows:

$$T.E. = \sqrt{\sigma^2 + \Delta Q^2}\,, \quad (1)$$

which is taken as the vertical error bar on the experimental data.

Mathematical Modeling

Continuity and Navier-Stokes equations

We model fluid flow through the ureteroscope by considering systematic reductions of the continuity and steady Navier-Stokes equations for an incompressible, viscous Newtonian fluid given by the following:

$$\nabla \cdot u = 0,\ (a) \quad \rho\left[\frac{\partial \boldsymbol{u}}{\partial t} + (\boldsymbol{u}\cdot\nabla)\boldsymbol{u}\right] = -\nabla p + \mu\nabla^2 u,\ (b) \quad (2)$$

where $\boldsymbol{u}$ is the fluid velocity, p is the pressure, t is time, and ρ and μ are the constant density and dynamic viscosity of the fluid, respectively. The reduced equations are solved using a combination of analytical and numerical techniques.

Straight scope model

We first consider fluid flow through the working channel of an isolated ureteroscope without considering the effects of the urological environment, or any impediments to the flow such as stone baskets or laser fibers. We assume that the flow is steady and remove the time-dependent term in Equation (2a). A schematic of the physical system under consideration is displayed in Supplementary Figure S4. The ureteroscope lies horizontally, and the irrigation fluid is fed to the working channel from an elevated reservoir at height h above the level of the scope. This column of fluid generates a pressure, P_{inlet} , at the inlet to the scope where the irrigation tubing is attached, which is higher than the pressure at the distal end, $P_{\text{intrarenal}}$, thus driving flow through the scope.

The ureteroscope working channel is depicted in Supplementary Figure S5. The hollow region only comprises a small portion of the scope, as space on the tip is required for

Supplementary Table S2. Table of Tools Used for Working Tool Experiments

Working tools	*External diameter*
Flexiva 200 laser fiber	0.0443
Flexiva 365 laser fiber	0.0604
OptiFlex basket	0.0433
Zero Tip basket	0.0633
ZIPwire guidewire	0.0965

All manufactured by Boston Scientific.

auxiliary features, such as the light and camera necessary for visualization. The relevant portion of the scope is modeled as a cylindrical channel with circular cross section, radius a, and length L, and we adopt an axisymmetric cylindrical polar coordinate system with axes r and z oriented as shown in Supplementary Figure S5. We consider unidirectional, axisymmetric flow corresponding to an axial velocity, w. By continuity, Equation (2a), w only depends on r. The Reynolds number determines the relative importance of inertial to viscous effects. For pipe flow, the Reynolds number is $\mathrm{Re} = \rho Qa/(\mu A)$, where a is the radius of the channel, Q is the volumetric flow rate, and A is the cross-sectional area. For typical parameters of the setup and volumetric flow rates calculated experimentally (Experiment Details section), we obtain $\mathrm{Re} \sim O(10^2) - O(10^3)$. In long, thin geometries, the size of the reduced Reynolds number, $(a/L)^2\mathrm{Re}$, determines the significance of inertial terms relative to viscous terms. For ureteroscope working channels, $a/L \approx 0.001$, giving a reduced Reynolds number of $O(10^{-4})$, and hence, we neglect inertial terms in the governing equations. Under these assumptions, Equations (2a and 2b) reduce to the following ordinary differential equation for the axial velocity, $w(r)$,

$$\frac{1}{r}\frac{\mathrm{d}}{\mathrm{d}r}\left(r\frac{\mathrm{d}w}{\mathrm{d}r}\right) = \frac{1}{\mu}\frac{\Delta P}{L}, \tag{3}$$

where ΔP is the pressure difference between the inlet and outlet of the scope ($\Delta P = P_{\mathrm{inlet}} - P_{\mathrm{intrarenal}}$ in Supplementary Fig. S4). We solve Equation (3) analytically, applying a no-slip boundary condition, $w = 0$, at $r = a$, and a symmetry condition $\mathrm{d}w/\mathrm{d}r = 0$ at $r = 0$, to obtain the solution for axial velocity through the working channel as a function of r.

$$w(r) = \frac{\Delta P}{4\mu L}(r^2 - a^2). \tag{4}$$

We integrate Equation (4) over a cross section to calculate Q, the total amount of fluid passing through a cross section per unit time to get the well-known Poiseuille relationship.

$$Q = \frac{\pi a^4 \Delta P}{8\mu L}. \tag{5}$$

Approximating P_{inlet} as a hydrostatic pressure gives $P_{\mathrm{inlet}} = \rho gh$, where h is the height of the column of fluid. However, as the fluid is, in actuality, not stationary within the irrigation tubing, $P_{\mathrm{inlet}} < \rho gh$, and we calculate the correction to hydrostatic pressure in the following section.

Viscous dissipation

Viscous dissipation due to the flow of fluid through the irrigation tubing results in the inlet pressure P_{inlet} being less than its hydrostatic approximation ρgh. The tubing is modeled as a cylindrical channel of radius R, oriented vertically, and we assume that the column of fluid, flowing due to gravitational forces, is maintained at height h (Supplementary Fig. S6). We assume that the top of the column of fluid is at the same pressure as the scope outlet (atmospheric pressure). The ratio of radius to length of the tubing is small, $R \ll L$, so that we can again use Poiseuille's law to relate flow through the tube to the pressure drop across it, as in Straight Scope Model section. The flow rate through the tube, Q_R, is then

$$Q_R = \frac{\pi R^4(\rho gh - P_{\mathrm{inlet}})}{8\mu h}, \tag{6}$$

where g is the acceleration due to gravity. As the fluid is incompressible, and assuming that both the tubing and working channel are rigid, then we must have that $Q_R = Q$ (Supplementary Fig. S6).

For flow through a straight scope with no working tool, we calculate ΔP by equating expressions (5) and (6) to get

$$\Delta P = \rho gh\left(1 - \frac{\sigma}{1+\sigma}\right), \tag{7}$$

where $\sigma = (h/L)(a/R)^4$ characterizes the relative importance of dissipative effects. In subsequent sections, we use this approach to include the effects of viscous dissipation.

Deflected scope model

When the scope is deflected during ureteroscopic procedures, the majority of the scope remains straight, while the tip can bend up to 270° in both directions (Supplementary Fig. S2). We model the deflected scope as a straight pipe, connected to a shorter pipe with constant radius of curvature R_{curv} (Supplementary Fig. S7). In the straight section, the volumetric flow rate is related to the pressure drop by Poiseuille's law, while in the curved section we determine the relationship between flow rate and pressure drop using the Dean flow equations, detailed in Dean.[S1]

We denote the unknown pressure where the scope transitions from straight to curved as P_{mid}, and the lengths of the straight and curved sections of the scope as L_s and L_c, respectively (Supplementary Fig. S7a). Assuming we know the pressures at the inlet and outlet of the scope, we can equate the flow rates through the straight and curved portions to determine the two unknown quantities, P_{mid} and the flow rate Q. To describe the flow through the curved section of the scope, we follow the formulation in Dean,[S1] assuming that the flow is steady and that $\delta = a/R_{\mathrm{curv}} \ll 1$. This is consistent with the values considered, as the minimum value for R_{curv} is 1 cm (see Supplementary Fig. S2), corresponding to a maximum $\delta = 0.06$.

The Dean equations[S1] involve a single dimensionless parameter, the Dean number.

$$D = \frac{\Delta P_c \rho a^3 \sqrt{2\delta}}{L_c \mu^2}, \tag{8}$$

where in our setup

$$\Delta P_c = P_{\mathrm{mid}} - P_{\mathrm{intrarenal}} \tag{9}$$

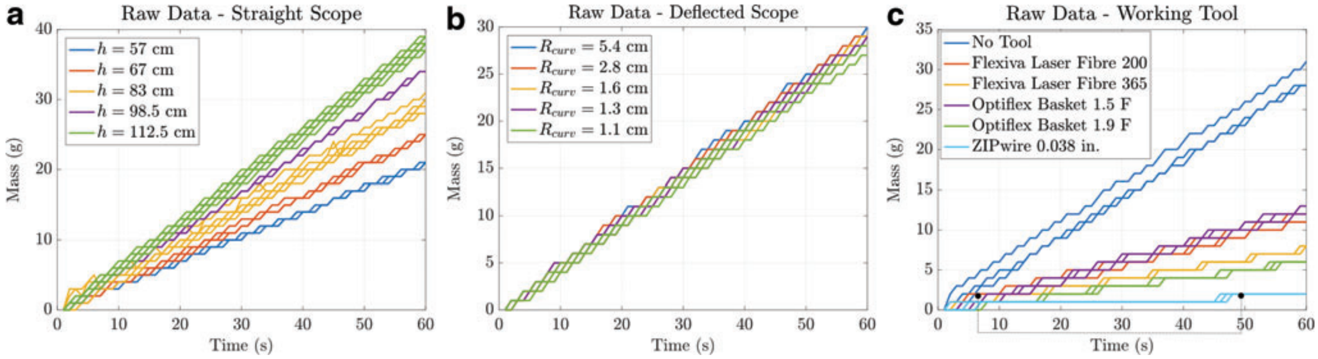

SUPPLEMENTARY FIG. S3. Experimental data for **a**. Straight scope, **b**. Deflected scope, and **c**. Scope with working tool. The vertical axis gives the mass of water in the collecting bucket in grams, and the horizontal axis is time in units of seconds.

is the pressure difference along the curved portion of the scope. For D small, the volumetric flow rate through the curved section is approximately[S1]

$$Q_c \approx \frac{\pi D a \nu}{8\sqrt{2\delta}}\left[1 - 0.0306\left(\frac{D}{96}\right)^4\right], \quad (10)$$

an expression that has been shown to be valid for Dean numbers up to approximately $D = 96$. Using a combination of asymptotic and numerical methods, Collins and Dennis[S2] computed a relationship between flux and D that is valid in the range of $D = 96$ to $D = 5000$, which is

$$Q_c \approx \frac{\pi D a \nu}{8\sqrt{2\delta}}\left[8.12 D^{-\frac{1}{3}} - 16.7 D^{-\frac{2}{3}}\right]. \quad (11)$$

To apply Equation (10) or (11) to compute the flux through the curved portion of the scope, we must first calculate the Dean number via Equation (8). This involves knowing ΔP_c and hence P_{mid}, which we calculate as a function of P_{inlet} and $P_{\text{intrarenal}}$, by equating the fluxes through the straight and curved portions of the scope, Q_s and Q_c, respectively (Supplementary Fig. S7c). Assuming that flow through the straight portion of the ureteroscope is governed by Poiseuille flow, we have that

$$Q_s = \frac{\pi a^4 (P_{\text{inlet}} - P_{\text{mid}})}{8 L_s \mu}, \quad (12)$$

and equating (12) with (10) or (11) we have the relationships

$$\frac{P_{\text{inlet}} - P_{\text{mid}}}{L_s} - \frac{P_{\text{mid}} - P_{\text{intrarenal}}}{L_c}\left[1 - 0.0306\left(\frac{D}{96}\right)^4\right] = 0, \quad \text{for } D \le 96, \quad (13)$$

$$\frac{P_{\text{inlet}} - P_{\text{mid}}}{L_s} - \frac{P_{\text{mid}} - P_{\text{intrarenal}}}{L_c}\left[8.12\, D^{-\frac{1}{3}} - 16.7 D^{-\frac{2}{3}}\right] = 0, \quad \text{for } D \ge 96, \quad (14)$$

which, because D depends linearly on P_{mid}, are each quintic equations to solve for P_{mid}. Once P_{mid} has been calculated, it can be used to compute the flux through the curved scope using Equation (12). However, because we require P_{mid} to compute D, it is not known whether to use Equation (13) or (14) beforehand. Thus, to solve for the flux for a given curvature ratio, δ, we solve both Equations (13) and (14) for P_{mid}, and then, after computing the Dean number, select the appropriate result.

As described in the Viscous Dissipation section, a more accurate estimation of the flow rate is obtained by including the effects of viscous dissipation in addition to the hydrostatic head. In this study, this is achieved by equating the flow through the vertical tubing, Equation (6), with the flow through the straight portion of the scope, Equation (12). We thus have a system of two equations for the unknown pressures P_{inlet} and P_{mid}, which we solve using MATLAB's fzero solver.

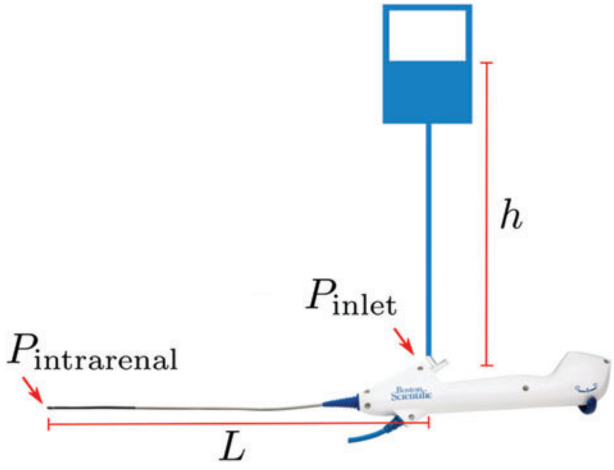

SUPPLEMENTARY FIG. S4. A schematic of the assumed setup when modeling flow through the ureteroscope working channel.

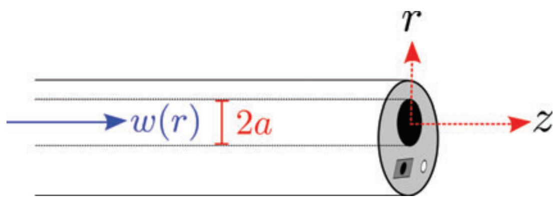

SUPPLEMENTARY FIG. S5. A schematic of the scope tip with a cylindrical polar coordinate system, where the z-axis is along the length of the scope and r is the radial coordinate. The working channel has radius a and we derive the velocity $w(r)$, in Equation (4).

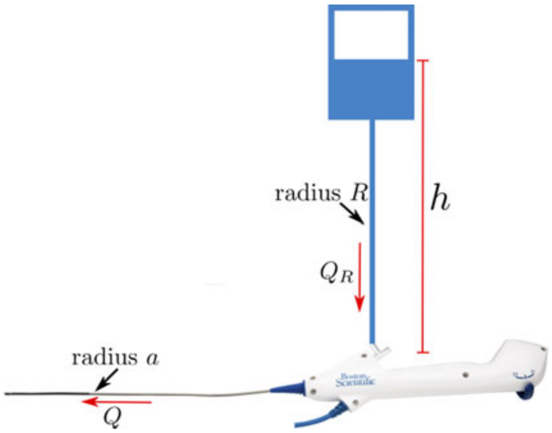

SUPPLEMENTARY FIG. S6. A schematic of the assumed setup, where fluid flows vertically through tubing of radius R into the working channel of the scope with radius a. The respective flow rates Q_R and Q_a must be equal by conservation principles.

We note from Equation (12) that the model also predicts a linear decrease in flow rate with increasing length of the curved portion. In practice, there is little room for varying the length of the deflectable tip; nevertheless, the theoretical approach implemented here may be useful in a more refined model to account for the effect of other scope curvatures along the length of the scope, for example, due to passage through the bladder and urethra.

Channel with working tool

Circular working tool. If the tool is concentric within the channel (Supplementary Fig. S8) where a is the radius of the working channel (outer circle) and b is the radius of the tool (inner circle), we can apply an exact solution for fully developed laminar flow in an annular duct. The axial velocity $w(r)$, which depends on both b and a, as well as ΔP, μ, and L, can be obtained by solving Equation (3), applying no-slip conditions on the inner and outer boundaries. The flux, Q, is obtained by integrating the velocity over the annular cross section, giving

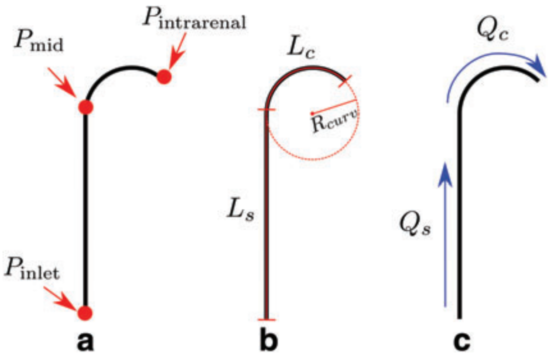

SUPPLEMENTARY FIG. S7. An idealized depiction of a deflected scope. **(a)** The three scope pressures, **(b)** the constant lengths of the curved and straight portions, and **(c)** the fluxes through each section, which must be equal by conservation principles.

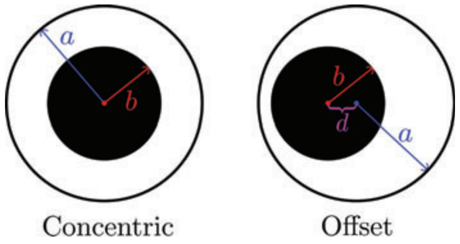

SUPPLEMENTARY FIG. S8. *Left*: schematic of the cross-sectional domain of a working channel with a working tool sitting in the center (concentric). The channel has radius a and the tool has radius b. *Right*: cross section of the same tool and channel, but with centers of the two circles a distance d apart (offset).

$$Q = \frac{\pi\left(a^2-b^2\right)\Delta P}{8\mu L}\left[a^2+b^2-\frac{a^2-b^2}{\log\left(\frac{a}{b}\right)}\right]. \tag{15}$$

From Equation (15) we see that as the radius of the tool increases, the channel is increasingly obstructed, with the flow rate going to zero as $b \to a$. If the tool is offset within the channel (Supplementary Fig. S8), it is no longer beneficial to model the system in polar coordinates, as the cross section is no longer axisymmetric. The governing equation for fully developed laminar flow[S3] is given in Cartesian coordinates (x, y) as follows:

$$\frac{\partial^2 w}{\partial x^2}+\frac{\partial^2 w}{\partial y^2}=\frac{1}{\mu}\frac{\Delta P}{L}, \tag{16}$$

where $w(x, y)$ is again the axial velocity, satisfying no-slip conditions $w = 0$ on the tool and channel boundaries. To solve for w, we implement bipolar coordinates that map offset circles to a rectangular domain. The mathematical details of this approach, as well as the methods used to solve the transformed equation, are presented in Williams et al.[S4]

Elliptical working channel. When considering an elliptical channel with a circular tool, we use finite elements in an open-source finite element library, oomph-lib, to solve Equation (16) for a specified pressure gradient. The elliptical shape and tool position that produce maximal flow are found by performing sweeps over relevant parameters, keeping the cross-sectional area available for fluid flow constant.

Including viscous dissipation. We include the effects of viscous dissipation through the irrigation tubing by equating the vertical flow through the tubing with the flow through the scope to solve for P_{inlet} (Viscous Dissipation section). The form of Equation (16) allows us to deduce that the flux through the tube with a working tool is proportional to ΔP

$$Q = \alpha \Delta P, \tag{17}$$

where α is dependent on the geometry of the domain and is calculated from our numerical simulations by solving Equation (16) subject to $\Delta P = 1$. Equating flow rate through the scope with the vertical flow Q_R [Equation (6)], we obtain

$$P_{\text{inlet}} = \frac{\pi R^4 \rho g h}{\alpha 8 \mu h + \pi R^4}. \tag{18}$$

We can thus insert this into Equation (17) to determine the flux, accounting for viscous dissipation effects.

Supplementary References

S1. Dean WR. LXXII. The stream-line motion of fluid in a curved pipe (SECOND paper). Lond Edinb Dublin Philos Mag J Sci 1928;5:673–695.

S2. Collins WM, Dennis SCR. The steady motion of a viscous fluid in a curved tube. Q J Mech Appl Math 1975;28:133–156.

S3. Lamb H. Hydrodynamics. Fetter Lane E.C.: Cambridge University Press, 1916.

S4. Williams JG, Turney B, Moulton DE, Waters S. The effect of geometry on flow resistance in coaxial elliptic cylinders. J Fluid Mech 2018. (In preparation).
